# Tracking Membrane Protein Association in Model Membranes

**DOI:** 10.1371/journal.pone.0005035

**Published:** 2009-04-01

**Authors:** Myriam Reffay, Yann Gambin, Houssain Benabdelhak, Gilles Phan, Nicolas Taulier, Arnaud Ducruix, Robert S. Hodges, Wladimir Urbach

**Affiliations:** 1 Laboratoire de Physique Statistique, École Normale Supérieure, UMR 8550 CNRS-UPMC, Université Paris 06, Paris, France; 2 UPMC Université Paris 06, UMR 7623, Laboratoire d'Imagerie Paramétrique, Paris, France; 3 CNRS, UMR 7623, LIP, Paris, France; 4 Laboratoire de Cristallographie et R.M.N. Biologiques, Université Paris Descartes, UMR8015 CNRS, Paris, France; 5 Department of Biochemistry and Molecular Genetics, University of Colorado at Denver, Denver, Colorado, United States of America; Dalhousie University, Canada

## Abstract

Membrane proteins are essential in the exchange processes of cells. In spite of great breakthrough in soluble proteins studies, membrane proteins structures, functions and interactions are still a challenge because of the difficulties related to their hydrophobic properties. Most of the experiments are performed with detergent-solubilized membrane proteins. However widely used micellar systems are far from the biological two-dimensions membrane. The development of new biomimetic membrane systems is fundamental to tackle this issue.

We present an original approach that combines the Fluorescence Recovery After fringe Pattern Photobleaching technique and the use of a versatile sponge phase that makes it possible to extract crucial informations about interactions between membrane proteins embedded in the bilayers of a sponge phase. The clear advantage lies in the ability to adjust at will the spacing between two adjacent bilayers. When the membranes are far apart, the only possible interactions occur laterally between proteins embedded within the same bilayer, whereas when membranes get closer to each other, interactions between proteins embedded in facing membranes may occur as well.

After validating our approach on the streptavidin-biotinylated peptide complex, we study the interactions between two membrane proteins, MexA and OprM, from a *Pseudomonas aeruginosa* efflux pump. The mode of interaction, the size of the protein complex and its potential stoichiometry are determined. In particular, we demonstrate that: MexA is effectively embedded in the bilayer; MexA and OprM do not interact laterally but can form a complex if they are embedded in opposite bilayers; the population of bound proteins is at its maximum for bilayers separated by a distance of about 200 Å, which is the periplasmic thickness of *Pseudomonas aeruginosa*. We also show that the MexA-OprM association is enhanced when the position and orientation of the protein is restricted by the bilayers. We extract a stoichiometry for the complex that exhibits a strong pH dependance: from 2 to 6 MexA per OprM trimer when the pH decreases from 7.5 to 5.5.

Our technique allows to study membrane protein associations in a membrane environment. It provides some challenging information about complexes such as geometry and stoichiometry.

## Introduction

Protein association is involved in a large array of biological processes: ligand-receptor interactions associated with cellular response to its environment, trafficking through export and fusion proteins, and antibiotic resistance mechanisms induced by efflux pumps [1]–[4]. If the detection of interactions between proteins constitutes the first step to characterize potential association, the most challenging and interesting problems remain the determination of the stoichiometry and the conformation of the protein complex. There is a plethora of techniques allowing the study of protein association such as Quartz Crystal Microbalance [5], Surface Plasmon Resonance [6], Blue Native Page [7], [8], ultracentrifugation [9]–[11] and structural biology. But none of these techniques can probe the interactions between membrane or transmembrane proteins as well as their organization in complex assemblies. So far, most techniques cannot do better than probing interactions between a membrane protein embedded into a bilayer and another protein solubilized in micelles. An exception is the Surface Force Apparatus technique. However these methods are using functionnalized supported bilayers that considerably restrict membrane movements and protein mobility [12]. Consequently, it is difficult to access the geometry of freely moving membranes on transmembrane protein association.

We develop an original approach to characterize the association between membrane or transmembrane proteins embedded within the same membrane or located in different membranes. Our approach can provide the conditions inducing the association (e.g. on membrane properties, pH…), the geometry and the stoichiometry of the protein association. Moreover our technique uses a two-dimensionnal environment close to the biological geometry.

### Principles of the experimental approach

The first step is to incorporate membrane proteins inside bilayers whose separation can be controlled at will in a kind of “soft” Surface Force Apparatus geometry. To do so, we use a system of model bilayers, called “sponge” phase (or L_3_) [13], some of which have been successfully used to crystallize membrane protein [14]. This phase consists of a randomly connected, continuous membrane. The L_3_ phase arises when the L_α_ (or lamellar) phase, comprised of alternating bilayers, is sufficiently dilute so that long-range positional and orientational (smectic) order is lost, but a bilayered membrane remains intact [15]–[17]. A transition from L_3_ to L_α_ phase can also be obtained by shear experiments [18]. The proposed L_3_ structure consists of locally sheetlike sections of semi-flexible surfactant bilayers, connected up at larger distances into a multiple connected random surface [19]. The radius of curvature R of a sponge phase can be approximated by 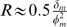 [20] where *φ_m_* is the membrane volume fraction and *δ_m_* designs the bilayer thickness. In our sponge phase, made of aqueous solvent, a non ionic surfactant (penta-monododecylether C_12_E_5_) and a co-surfactant (*β*-octylglucoside *β*-OG), the bilayer thickness is *δ_m_* = 32 Å and the bilayer volume fraction is in the range 0.03<*φ_m_*<0.08 thus the radius of curvature is very large 0.2 µm<R<1.6 µm. In addition we calculate the persistence length of the bilayer *ξ_K_* defined as [21]: 
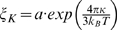
 where *a* is a molecular size of the order of 10 Å, *κ* is the mean curvature rigidity modulus. In our case, *κ* is in the range of a few *k_B_T* [22] leading to a persistance length around 1 µm, larger than the size of a membrane protein. Thus locally (around the protein) L_3_ looks like the disoriented lamellar phase over distance of the persistance length.

In such a system, the bilayer separation, precisely adjusted by adding the correct amount of aqueous solvent to the sample [23]–[25], is checked by Small Angle X-ray Scattering (SAXS) measurements (Supplementary data figure S1). Sponge phase gives a broad peak which position is related to the spacing between bilayers and the width to the thermal position fluctuations and the random pore structure. Moreover the asymptotic behavior at small *d* (or at large scattering vector) is described by a *d^2^* variation law as expected for a local bilayer structure [23].

The choice of a sponge phase lies in the fact that C_12_E_5_ and *β*-OG are extensively used for membrane and transmembrane protein solubilization and crystallography [26]–[29] and in particular for the OprM and MexA proteins studied in this article. However the approach described here can be applied with any sponge phase. The use of a non-ionic surfactant instead of lipids ensures the stability of the L_3_ system over a wider range of separation distances (from 50 to 500 Å), temperatures (from 6 to 30°C), ionic strength, and pH [30]. This phase allows the insertion of a wide range of membrane objects (peptides, anchored lipids and transmembrane proteins) [29] (see Materials ands Methods). The capacity of the sponge phase to retain the biological activity of the transmembrane protein bacteriorhodopsin was previously demonstrated [31].

**Figure 1:**
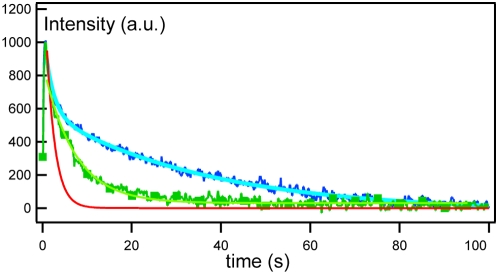
Example of recovery signals. The graph represents the fluorescence recovery signals obtained for differents molar ratios *r* = [B-L_12_]/[S]: *r* = 0 (red solid line), *r* = 0.2 (green squares), *r* = 1.9 (blue solid line). These signals are well-fitted by a single exponential law for *r* = 0 (corresponding to *D_0_* = 50 µm^2^ s^−1^), a double exponential for *r* = 0.2 (corresponding to *D_0_* = 50 µm^2^ s^−1^ and *D_1_* = 3.2 µm^2^ s^−1^), a triple exponential for *r* = 1.9 (related to *D_0_* = 50 µm^2^ s^−1^ , *D_1_* = 3.2 µm^2^ s^−1^ and *D_2_* = 1.6 µm^2^ s^−1^.

We use the Fluorescence Recovery After fringe Pattern Photobleaching (FRAPP) technique [32] to measure the recovery of fluorescence intensity, *I(t)*, of the signal emitted by FITC (Fluoresceine IsoThioCyanate)-labeled proteins inserted into the sponge phase. *I(t)* is well fitted by single or multiple exponentials 
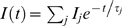
, where *τ_j_* is the recovery time characteristic of the diffusing fluorescent species *j* (Figure 1). Recovery times are used to derive the diffusion coefficients, *D_j_*, of each protein species, *j*. It has been recently demonstrated that the diffusion coefficient of diffusing molecules within a two-dimensional surface is proportional to their size according to the Stokes-Einstein like equation [29]: 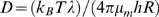, where *R* is the radius of the diffusing molecule or assembly of molecules, *h* the membrane thickness, *μ_m_* the membrane viscosity, *λ* a characteristic length related to enhanced membrane dissipation [33], *k_B_* the Boltzmann constant, and *T* the temperature. Consequently, when studying a protein of known size, we can readily determine if the protein is effectively embedded within the membrane (the diffusion coefficient on a two-dimensional surface should be an order of magnitude smaller than the same object in three dimensions), and if it is a monomer, dimer, or wether it forms a larger complex.

We focus our investigation on the interaction between two different transmembrane or membrane proteins. In our approach, the first step consists to determine the diffusion coefficient *D* of each protein species. The *D* value of a free fluorescently labeled protein is measured at a low concentration, in the absence of the other protein, and for a separation distance between bilayers *d_W_* that is very large. In this case, the *D* value is inversely proportional to its radius according to the above equation. The deduced radius compared with the crystallographic data gives the oligomerization state of the protein. Next, studies are performed in the presence of the two proteins where only the more mobile one is labeled. In such a way, any interaction will be reflected in a decrease of the diffusion coefficient of the labeled protein as any protein complex (that includes at least one labeled protein) will move slower than the free protein. Large *d_W_* values will permit to screen only lateral interactions between proteins embedded into the same bilayer. Interactions between proteins embedded in facing bilayers will be investigated by studying samples exhibiting a *d_W_* dependence. In these measurements, we will determine various diffusion coefficients: one will correspond to the free labeled protein (with a value identical in the absence or in the presence of the unlabeled protein) and the other ones to bound protein species. These measurements also provide the *d_W_* range at which protein interactions occur from facing bilayers. Once all diffusion coefficients have been ascribed to a protein species, concentration-dependent measurements allow us to extract the stoichiometry of the various observed complexes.

We have applied this approach to two systems. The first one is the well-known streptavidin-biotinylated peptide system and the second one is composed of two proteins from an efflux pump where little is known about their interactions.

## Results and Discussion

### Model system: the streptavidin-biotin association

In order to validate our approach, we study a model system: the association between the soluble protein streptavidin (S) and a biotinylated transmembrane peptide (B-L_12_), whose α-helix is made of twelve leucine residues. The binding of streptavidin to biotin is a very well characterized process [34]–[36]. Streptavidin protein is composed of four monomers, each containing a binding site for biotin. The general shape of streptavidin can be approximated as a parallelepiped, with two opposite sides (separated by 55 Å) each containing two binding sites [36], [37]. Furthermore, we have demonstrated in a previous work [29] that, after incorporation into the sponge phase, the transmembrane peptide was effectively embedded into the surfactant bilayer. Consequently, the streptavidin-biotin association represents a good model for the association between proteins embedded in the same bilayer or in facing ones. In this section, we show how to perform and interpret the well-designed FRAPP measurements to extract information about the protein-biotinylated peptide association.

### Functional distances for streptavidin-biotinylated peptide binding

In the following measurements, a sponge phase with a constant spacing distance between bilayers of *d_W_* = 120 Å is used.

**Figure 2:**
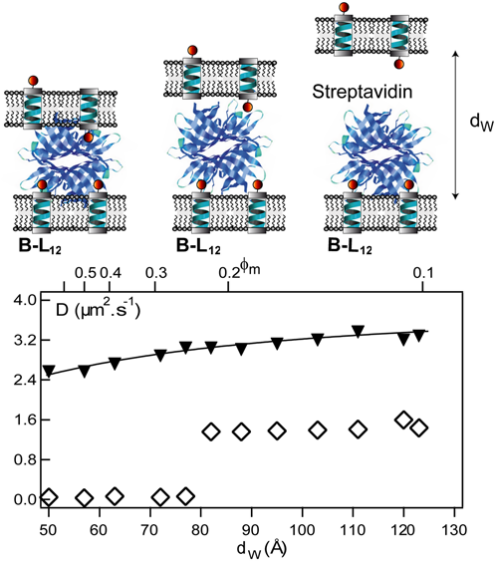
Evolution of the diffusion coefficient of bound streptavidin with the distance between bilayers. The top sketch represents a FITC-labeled streptavidin that is bound to two biotinylated peptides for large *d_W_* values, and that bridges peptides from two different bilayers when *d_W_*<80 Å as seen below for (empty diamonds). The bottom graph displays the diffusion coefficients, *D*, versus the separation distance between bilayers, *d_W_*. (black triangles down) represents the diffusion coefficient, D_L12_, of FITC-labeled L_12_ peptides embedded in the bilayers of the L_3_ phase devoid of streptavidin. This result is well described by the equation 1 (black solid line) as predicted for a surface diffusing object. (empty diamonds) are for FITC-labeled streptavidin proteins inserted in the sponge phase in the presence of biotinylated peptides, B-L_12_, at the molar ratio [B-L_12_]/[S] = 10. For *d_W_*>80 Å, streptavidins bind biotinylated peptides embedded in the same bilayers and for *d_W_*<80 Å, streptavidins bind biotinylated peptides embedded from opposite bilayers. Error bars are smaller than symbol size. The membrane volume fraction *φ_m_* corresponding to each size is indicated at the top of the graph.

The measurement of *I(t)* from FITC-labeled transmembrane peptides L_12_ in the sponge phase results in a single diffusion coefficient of *D_L12_* = 3.2 µm^2^ s^−1^ (Figure 2). The measurement performed on FITC-labeled streptavidins in the sponge phase also yields a single diffusion coefficient but with a larger value: *D_S_* = 50 µm^2^ s^−1^ (Figure 1). Since the longest dimension of streptavidin [36], [37]) is much smaller than the spacing distance *d_W_*, the value for *D_S_* should be characteristic of streptavidins that mainly freely diffuse between the bilayers of the sponge phase.

The investigation of the association between streptavidin and biotinylated peptides requires that the fastest diffusing molecule is labeled with FITC while the slowest one is kept unlabeled. Any binding between the two molecules will result in a drastic decrease in the diffusion coefficient of the labeled one. Therefore, *I(t)* is measured at a constant concentration (5.76 µM) of FITC-labeled streptavidin but with different concentrations of non-labeled and biotinylated L_12_ peptides, B-L_12_, added to the sponge phase.

For a molar concentration ratio [B-L_12_]/[S]<0.2, the recovery intensity is well fitted by a sum of two exponentials, 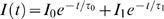, that provides two diffusion coefficients: *D_0_* = 50 µm^2^ s^−1^ and *D_1_* = 3.2 µm^2^ s^−1^. A straightforward comparison with the above values, *D_S_* and *D_L12_*, indicates that *D_0_* and *D_1_* are characteristic of a non-bound streptavidin and a streptavidin bound to a B-L_12_ peptide, respectively. It may appear surprising that a streptavidin bound to a B-L_12_ peptide has the same diffusion coefficient as a free L_12_ peptide. The reason originates from the much higher viscosity of the bilayer as compared to the surrounding aqueous medium [29], [38].

For 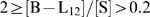, the analysis of the recovery signal leads to three diffusion coefficients (Figure 1): *D_0_* = 50 µm^2^ s^−1^, *D_1_* = 3.2 µm^2^ s^−1^, and *D_2_* = 1.6 µm^2^ s^−1^.

Finally, for 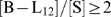, the recovery intensity is perfectly fitted by a single exponential 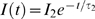 that leads to *D_2_* = 1.6 µm^2^ s^−1^. If we consider that the spacing distance between bilayers (≈120 Å) is large enough to prevent streptavidin from binding peptides embedded in facing bilayers, then *D_2_* should characterize the diffusion of streptavidin bound to two biotinylated peptides embedded in the same bilayer. To confirm this assumption we perform measurements for *d_W_* varying from 125 to 50 Å. When streptavidin is inserted into the sponge phase devoid of B-L_12_ peptides, we observe a single diffusion that smoothly decreases from 50 to 11 µm^2^ s^−1^ when *d_W_* decreases from 120 Å to 80 Å (data not shown). Below 80 Å the value stays constant to 11 µm^2^ s^−1^. We interpret these data as the effect of confinement due to bilayers proximity, which appears to be maximum for *d_W_*<80 Å, when streptavidin is entrapped in between the two surrounding bilayers. The variations are consistent with the model of Anderson and Wennerström [39] for hydrophilic object diffusion in a sponge phase, they scaled a linear variation with the membrane volume fraction *φ_m_* [40]. In the same phase, the diffusion coefficient of the transmembrane peptide is studied (Figure 2). Its diffusion coefficient decreases from 3.2 to 2.4 as *d_W_* decreases from 120 to 80 Å. This variation is well fitted by *φ_m_* ^2^:
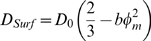
(1)where *D_0_* is the diffusion coefficient in a bilayer (in a lamellar phase) and *b* is related to the topology of the sponge phase. This law is characteristic of an object diffusing in the bilayer of the sponge phase (Figure 2) and has been succesfully used in several sponge phases [41].

Next, streptavidin is inserted in the sponge phase with an excess molar ratio, [B-L_12_]/[S] = 10, to ensure that all streptavidin proteins bind at least two B-L_12_ peptides (Figure 2) from the same bilayer. No change in the single diffusion coefficient is observed from *d_W_* = 120 to 80 Å and its value is of the order of the previously measured *D_2_*. Below 80 Å, it dramatically drops to 0.05 µm^2^ s^−1^ and maintains this value down to *d_W_* = 50 Å. At this concentration ratio and bilayer separation distance, we can reasonably assume streptavidin to bridge two bilayers by binding biotinylated peptides embedded in facing bilayers. The diffusion coefficient value (0.05 µm^2^ s^−1^) of a bilayer-bridging streptavidin is much smaller than for a confined streptavidin (11 µm^2^ s^−1^) and than *D_2_* ( = 1.6 µm^2^ s^−1^). Thus, this result confirms that *D_2_* is effectively reflecting a diffusion coefficient of a streptavidin bound to two peptides embedded into the same bilayer. We also observe that the bridging event appeared for *d_W_*<80 Å when streptavidin starts to be confined by two bilayers.

In conclusion, the concentration and *d_W_*-dependence measurements of the diffusion coefficient make it possible to discriminate between the free streptavidin and the peptide-bound streptavidins and to determine the characteristics of each species.

### Stoichiometry of streptavidin-biotinylated peptide complex

**Figure 3:**
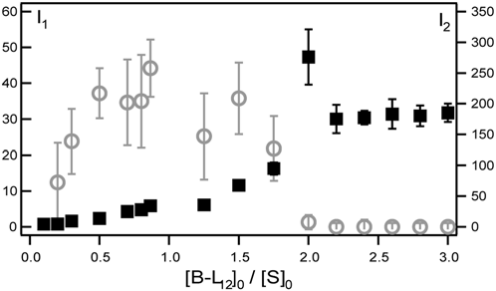
Determination of the number of interaction sites of the streptavidin: Variation of intensities of the free and bound streptavidin with the molar ratio r. The graph shows the behavior of intensities *I_1_* (grey circles) and *I_2_* (black squares) as a function of the molar ratio *r* = [B-L_12_]/[S]. *I_1_* represents the intensity of the signal corresponding to the diffusion coefficient *D_1_* = 3.2 µm^2^ s^−1^ whereas *I_2_* refers to the intensity of the signal corresponding to the *D_2_* = 1.6 µm^2^ s^−1^ diffusion coefficient. This graph reflects the stoichiometry of the corresponding proteins species. The first species is characterized by a small and broad peak centered around 1 that suggests that streptavidin is bound to one biotinylated peptide. The intensity of the second species increases as *r* increases to reach a plateau at *r* = 2 which suggests a stoichiometry of two biotinylated peptides per streptavidin.

For all our measurements, we keep an identical gain and detection sensitivity and use concentrations smaller than 60 µM for the diffusing molecules. By doing so, fluorescent intensities are proportional to concentrations of fluorescent-labeled molecules: the higher the intensity *I_j_*, the larger the population of protein species *j*. The stoichiometry is derived by plotting the behavior of the intensity of each protein species as a function of the molar ratio *r*, as shown in Figure 3. The intensity, *I_1_*, of the first species is characterized by a broad and very small peak centered approximately at *r* = 1. For *r*>2, we observe that *I_1_* = 0. The intensity *I_2_* of the second species increases with *r* at the same rate than *I_1_* for *r*<1. But *I_2_* continuously increases for 1<*r*<2, whereas *I_1_* decreases, and reaches its maximum at *r* = 2 when *I_1_* becomes null. These results reflect the non-cooperative binding of a streptavidin with biotinylated peptides embedded in the same bilayer surface. When the concentration of biotinylated peptides increases, the populations of streptavidins bound to one B-L_12_ peptide (species 1) and to two B-L_12_ peptides (species 2) simultaneously increase. When all streptavidins are bound to at least one peptide, the species 1 starts to disappear as these streptavidins are now binding a second peptides to form the species 2. The species 2 is at its maximum at the ratio *r* = 2 when no more binding occurs.

### Interaction between membrane proteins of an efflux pump

**Figure 4:**
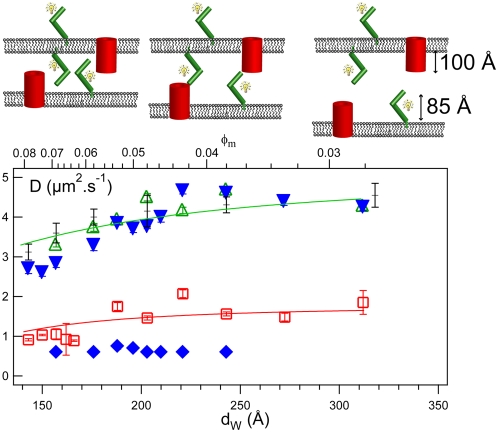
Interaction between MexA and OprM: Evolution of the diffusion coefficient of the proteins with the distance between bilayers. The top sketch represents OprM trimer (barrel) and MexA (sticks) protein diffusing in the bilayers. The bottom plot represents the variation of diffusion coefficients vs bilayer distance *d_W_*: for FITC-labeled MexA (green triangles up),FITC-C_16_ (a SOPC lipid) (black crosses) and OprM (red empty squares) in the sponge phase devoid of OprM and MexA, respectively, for free (blue triangles down) and bound (blue diamonds) MexA inserted in a sponge phase containing OprM trimers. The results are compared with the expected *φ_m_* ^2^ variation law (equation 1) for MexA (green solid line) and for OprM(red solid line). The only change compared with the peptide variation is the value of the asymptotic diffusion coefficient. Error bars are smaller than symbol size.The corresponding volume fraction *φ_m_* is indicated at the top of the graph.

*Pseudomonas aeruginosa* is a gram-negative bacterium known to be highly resistant to antibiotics. This resistance is believed to be partly related to efflux pumps expressed by *P. aeruginosa*. The function of these efflux pumps is to transport various molecules like antimicrobials out of the cell through a double membrane using a recently described mechanism [1]–[4]. The double membrane contains a periplasm whose thickness is close to 200 Å [42]. Twelve genetically distinct efflux pump assemblies (each composed of three proteins) are described in *P. aeruginosa* genome. The protein assembly MexA-MexB-OprM is the only one that is constitutively expressed, whereas the others are expressed under special circumstances. MexB [43] and OprM [44] are trimeric transmembrane proteins located at the inner and outer membrane, respectively. While both MexA and OprM are lipoproteins with a palmitoyl moiety, OprM is an integral transmembrane protein. The periplasmic component MexA is assumed to bridge MexB to OprM [43]–[48] (Figure 4). MexB is the only active part of the pump. The exact organization and mechanism of the efflux pump assembly MexA-MexB-OprM is still unknown. In particular, the exact degree of MexA oligomerization within a single efflux pump is unclear. The crystallographic structure of MexA leads to a model where six or seven MexA proteins are assembled in a horseshoe manner. Three different models for the MexB-MexA-OprM assembly suggest that 3, 6, or 9 MexA monomers could participate in the assembly, respectively [46]–[48]. Furthermore, it has not yet been determined if MexA interacts either with OprM or MexB or with both proteins. Finally, the anchoring of MexA to the membrane and its conformation when anchored has not been established [47]. In this article, we focus our study on the assembly between MexA and OprM proteins by using the approach described in the previous section. As MexA and OprM are not active in the efflux process, their functionnality is still unclear, the only avalaible data are crystallographic structures. To monitor the insertion of the two proteins inside the bilayers their secondary structure inside the sponge phase is compared with the one in their initial detergent β-OG (supplementary data Figure S3) and with their published crystallographic structures [44]–[46]. The Circular Dichroism (CD) spectra of either MexA and OprM proteins indicate that their general structure is similar between the protein in solution and in sponge phase. The quantities of α-helices and β-sheets measured from the spectra are also compatible with the crystallographic data of the proteins.

### Functional distances for MexA-OprM association

We study sponge phase samples with a spacing distance between bilayers, *d_W_*, ranging from 90 to 320 Å. Crystallographic data show that the hydrophilic domains of the rather rigid OprM and the rather flexible MexA are 100 Å and 85 Å long, respectively [44]–[46]. Furthermore, it has been proposed that OprM and MexA could dock together in the same way that has been proposed for TolC-AcrA [49].

When only FITC-labeled MexA bearing a palmitoyl moiety is inserted into the sponge phase, FRAPP measurements give a single diffusion coefficient, *D_MexA_*, whatever the distance *d_W_* (Figure 4). Interestingly, the behavior of *D_MexA_* is identical to that of *D_C16_* of a FITC-labeled SOPC lipid (FITC-C_16_) inserted in the sponge phase under the same conditions (Figure 4). The SOPC lipid and the MexA palmitoyl moiety possess the same aliphatic chain length. We also measure the diffusion coefficient of a mutant of MexA, called mMexA, in which the unique N-terminal cysteine is deleted (hence precluding the formation of the hydrophobic moiety) [47]. We observe that the diffusion coefficient of the soluble mMexA protein, *D_mMexA_* = 25 µm^2^ s^−1^, is about ten times larger than that of MexA protein. These results indicate that MexA is effectively anchored to the bilayers by its single sixteen-carbon chain and is in a monomeric state (otherwise *D_MexA_* would be larger than *D_C16_* according to [29]). The observed decrease in diffusion coefficient from 4.2 to 2.3 µm^2^ s^−1^ (Figure 4) is well described by the equation 1. The variation law is similar with the one found for the peptide B-L_12_ as expected for a diffusion inside the same phase [39], [41].

When FITC-labeled OprM is inserted into the sponge phase, a single diffusion coefficient *D_OprM_* is observed that follows a similar behavior to MexA except that OprM is much less mobile with *D_OprM_* = 1.4 µm^2^ s^−1^ for *d_W_*>170 Å. This value leads to a hydrodynamic radius of *R_OprM_* = 17±2 Å for the OprM trimer [27], in good agreement with crystallographic and electron microscopy data [44], [50], [51]. These results present the evidence that OprM is embedded into the surfactant bilayers of the sponge phase. The fit of OprM diffusion coefficient by equation 1 for *d_W_*>130 Å confirmed this anchorage. For *d_W_*<130 Å, a significative decrease of *D_OprM_* is observed, dropping to 0.1 µm^2^ s^−1^ at *d_W_*<100 Å, i.e. when *d_W_* is smaller that the OprM trimer hydrophilic length. So this last behavior reflects the hindrance of OprM mobility due to confinement.

The investigation of interactions between MexA and OprM trimer is performed by incorporating FITC-labeled MexA into a sponge phase containing unlabeled OprM. As MexA protein is more mobile than OprM trimer (i.e. *D_MexA_>D_OprM_*), any association between MexA and OprM will be reflected by a decrease in the diffusion coefficient of the FITC-Labeled MexA protein. The concentration of MexA (*C_MexA_* = 0.1 µM) is ten times smaller than that for OprM trimer (*C_OprM_* = 1 µM). We measure the behavior of the diffusion coefficient of MexA for *d_W_* ranging from 320 to 140 Å (see Figure 4 and Table 1).

**Table 1:** The first column gives the values of the separation distance between bilayers and the second one the molar ratio of MexA over OprM protein.

dw (Å)	r = [MexA]/[OprM]	Number of exponential	D_I_ (µm^2^/s)	D_II_ (µm^2^/s)
dw<240	0.1	1	D_MexA_	
140<dw<240	0.1	2	D_MexA_	≈0.6
dw<140	0.1	1	D_MexA_	

The number of exponentials used to fit the fluorescent intensities data is given in the third columns along with the values of each diffusion coefficient in the following columns. D_MexA_ is the value of the diffusion coefficients of a free labeled MexA measured in the absence of OprM. The value 0.6 µm^2^/s is characteristic of MexA bound to OprM from two facing bilayers.

For *d_W_*>250 Å, MexA exhibits a single diffusion coefficient value which experiences the same behavior as when no OprM is present into the sponge phase. This is evidence that no interaction takes place between MexA and OprM trimer, either laterally on the same bilayer or from two facing bilayers.

From *d_W_*≈250 Å to 150 Å, two diffusion coefficients are observed. The first coefficient is still equal to the value of *D_MexA_* found in the absence of OprM, the second one exhibits a value equal to 0.6 µm^2^ s^−1^, which is close to but smaller than *D_OprM_*. This last diffusion coefficient should be associated with MexA bound to OprM trimers. Indeed, the complex MexA-OprM trimer is moving with a slower mobility than the respective free protein and trimer. We also check that this value is independent of protein concentration, so we can rule out the possibility of aggregation.

For *d_W_*<150 Å, we again observe a single diffusion coefficient the value of which is similar to a free MexA diffusing in a sponge phase devoid of OprM.

**Figure 5:**
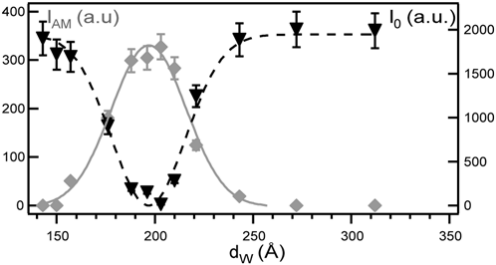
Determination of the best interaction condition : Evolution of bound MexA with the distance between bilayers. Variation of fluorescence intensities *I_0_* and *I_AM_* of free MexA (black triangles down) and MexA bound to OprM trimers(grey diamonds), respectively. Since fluorescence intensity is proportional to a MexA species population, we observe a shift in population from the free to the OprM-bound MexA species as the spacing distance approaches *d_W_* = 200 Å. At *d_W_*≈200 Å, almost all MexA proteins are bound to OprM trimers. These data are fitted by two gaussian curves.

From these results, we conclude that MexA and OprM trimer can interact only when embedded within two facing bilayers and only when these bilayers are separated by a distance of 150<*d_W_*<250 Å. We observe in figure 5 that the population of bound MexA-OprM is at its maximum at *d_W_*≈200 Å. Interestingly, this value is similar to the periplasm thickness of *P. aeruginosa*. Our results are in favor of the docking sites being located at the end of the hydrophilic periplasmic loops of OprM trimer and on the α-helical hairpin of MexA [44], [45], [52].

The intensity variations of free and bound MexA versus *d_W_* are well fitted by gaussian curves 
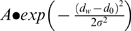
 where the fits give σ≈21 Å (Figure 5). These behaviors reflect the fact that, due to bilayers fluctuations, the bilayer separation is not everywhere equal to *d_W_*, the mean distance obtained from SAXS.

For comparison, we perform analogue experiment at *d_W_*≈200 Å with mMexA, the soluble mutant of MexA. We observe that *D_mMexA_* drops from 28 µm^2^ s^−1^ to 1.4 µm^2^ s^−1^ which is, this time, identical to the diffusion coefficient of a free OprM trimer. This brings evidences that mMexA binds the OprM trimer and that there is no “bridging effect”. Moreover, the intensity due to the bound fraction of mMexA protein increases and reaches a steady state only one hour after the beginning of the experiment, as compared to the short delay of two minutes for MexA protein. This result suggests that the orientation and anchoring of MexA protein to the membrane is a key condition for the dynamic of the interaction.

### Stoichiometry of the MexA-OprM complex

In order to determine the stoichiometry of the MexA-OprM complex, we use the sponge phase at *d_W_*≈200 Å, which is the prerequisite for an optimal MexA to OprM trimer binding, as shown above. Samples are prepared with different molar ratios, *r*, of FITC-labeled MexA protein to unlabeled OprM trimer. The concentration of OprM trimer is kept constant to *C_OprM_* = 1 µM. The aqueous solution of the sponge phase has a pH value of 7.5. For all molar ratios, FRAPP measurements give two diffusion coefficients *D_I_* and *D_II_*. *D_I_* is equal to the diffusion coefficient, *D_MexA_*, of a free MexA protein in the sponge phase, whereas the value of *D_II_* is equal to *D_MexA-OprM_* = 0.6 µm^2^ s^−1^ which is associated with MexA proteins bound to OprM trimers, as previously shown (Table 1).

**Figure 6:**
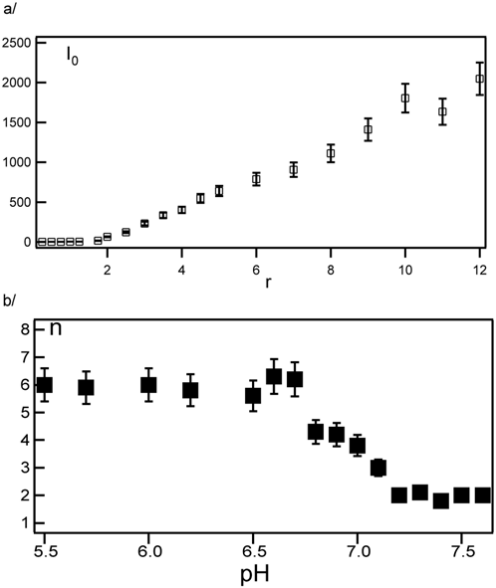
Determination of the stoechiometry of the complex MexA-OprM: Evolution of the intensities of free MexA with the molar ratio r and the evolution of the stoichiometry with pH. (a) Variation of the intensity *I_0_* associated to free FITC-labeled MexA proteins as a function of molar ratio *r*. In this experiment, *d_W_* = 200 Å and pH = 7.5. This variation provides the stoichiometry number equal to n_MexA_/n_OprM_ = 2. (b) Variation of the stoichiometry number with pH.

The stoichiometry of the MexA-OprM complex is derived by monitoring the behavior of the fluorescence intensity variation. A quenching phenomenon appears for *I_AM_*, associated with OprM-bound MexA proteins, that is probably due to a close proximity between MexA proteins. This effect prevents the use of *I_AM_* in the determination of the stoichiometry. We consider the variation of the fluorescence intensity *I_0_* associated with free MexA (Figure 6a). For *r*<2 the intensity *I_0_* is almost null, indicating that most MexA proteins are bound to OprM. For *r*>2, the linear behavior of *I_0_* with MexA concentration suggests that no more MexA proteins bind to OprM, since all new additions of MexA contribute to *I_0_*. Consequently, under the experimental conditions the variation of *I_0_* suggests a stoichiometry of two MexA proteins bound to one OprM trimer at pH 7.5. The pH-dependence of the stoichiometry has been investigated and the result is reported in Figure 6b where the stoichiometry increases from 2 to 6 when the pH decreases from 7.5 to 5.5. This is in agreement with blue native gel experiments (to be published) and with immuno-blotting experiments [53]. From these results, we conclude that the number of binding sites on each OprM trimer is governed by acid-base equilibria.

pH-dependent conformational changes of AcrA, a protein analogous to MexA within the AcrA/B TolC *E. coli* efflux pump have been reported [54] and a similar behavior can be presumed for MexA. Our result correlates these observed conformation changes with a variation in the stoichiometry of the interaction with OprM.

### Conclusion

An original approach is developed combining the FRAPP technique and the use of a versatile L_3_ sponge phase that makes it possible to extract crucial information about interactions between membrane proteins embedded in the bilayers of the sponge phase. The clear advantage lies in the ability to adjust at will the spacing between two adjacent bilayers. After validating our approach on the streptavidin-biotinylated peptide complex, we study the interactions between two membrane proteins, MexA and OprM, from bacterial efflux pump. The mode of interaction, the size of the protein complex and its potential stoichiometry are determined. In particular, we demonstrate that: MexA is effectively embedded in the bilayers; MexA and OprM interact only if they are embedded in opposite bilayers separated by a distance close to the periplasmic thickness in *P. aeruginosa*. We also show that the MexA-OprM association is enhanced when the position and orientation of the protein is restricted by the bilayers. The stoichiometry of the complex exhibits a strong pH dependence in the range 2 to 6 MexA per OprM trimer. Our approach can be extended to different transmembrane protein complexes that are difficult to investigate by other methods. The method is not limited by the number of proteins so the association of several proteins can be studied in the same way. It can also be expanded to other areas, opening new avenues: for example to co-crystallize proteins or to study protein-DNA interactions by screening the experimental conditions governing the complex formation.

## Materials and Methods

### Proteins, peptides and probes

FITC (Fluoresceine IsoThioCyanate)-labeled streptavidin was purchased from Interchim and used as received. The twelve-leucine α-helix transmembrane peptide (L_12_) whose sequence is AKK-(L)_12_-GKK was synthesized and biotinylated (B-L_12_) in the Department of Biochemistry and Molecular Genetics (University of Colorado, Denver). FITC-hexadodecylcarbonyl (FITC-C_16_) was purchased from Molecular Probes and used as received. The periplasmic protein MexA was expressed and purified according to [55] in its mature form and in a mutant form (with the N-terminal cystein deleted) [47]. The outer membrane protein OprM was expressed and purified as previously described [50]. Depending of the experiments, OprM and MexA could be labeled with FITC (Molecular Probes). All proteins were solubilized in a solution *S_1_* of composition: 1% (w/w) β-octylglucoside (β-OG) (from Sigma), 100 mM NaCl, 50 mM Na_2_HPO_4_ pH 7.5, and 5% (v/v) glycerol. The L_3_ phase [56], [57] was prepared by mixing altogether a non-ionic surfactant [penta-monododecylether (C_12_E_5_, from Nikko Chemicals- Jan Dekker)], a co-surfactant β-OG (molar ratio [C_12_E_5_] /[β -OG] = 7) and an aqueous solution (100 mM NaCl, 50 mM Na_2_HPO_4_ pH 7.5, 5% (v/v) glycerol). In such a system, although bilayers are randomly connected, a mean distance between bilayers can be locally defined. A volume of solution *S_1_*, containing the proteins, was then added and mixed with the sponge phase so that the C_12_E_5_ to β-OG molar ratio equals 7. The membrane volume fraction *φ_m_* is defined by the ratio 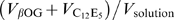. The separation distance between bilayers can be very precisely tuned by simply varying *φ_m_*. The L_3_ phase is prepared within a minute and remains stable at least for several months. The protein concentration in the L_3_ phase was controlled during the sample preparation. In this study, measurements were performed on individual samples for each protein concentration and bilayer separation distance.

### Small Angle X-ray Scattering (SAXS) technique

The separation distance between bilayers, (*d_W_*), has been determined using small angle X-ray scattering (SAXS) technique. Measurements were performed using a rotating anode generator. The Cu-K_α1_ wavelength (1.54 Å) was selected by a gold-coated quartz mirror. The scattering intensity was recorded as a function of the scattering vector *q* 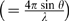 using a detector with a spatial resolution of 0.2 mm. The distance from the detector to the sample was 770 mm. The final resolution of the set-up was 0.02 nm. Samples were placed in sealed glass capillaries and positioned in a thermostated holder (*T* = 296 K). All spectra exhibited a broad peak indicating that the material has a well defined characteristic spacing (Supplementary data figure S1) with an intensity decay scaling asymptotically as *d^2^* with 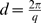 for small *d* resulting from the bilayer structure [56]. The peak position provided the characteristic distance 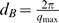 with a precision of 5%. Sponge phases have also been observed by freeze-fracture electron microscopy (Supplementary data figure S2) and the distance between bilayers extracted from these micrographs matches the values obtained by SAXS [57]. The separation distance between bilayers, *d_W_*, was deduced by subtracting the bilayers thickness *δ_m_* = 32 Å [58] to *d_B_* which is kept constant. The asymptotic behavior is checked in the insert of the supplementary figure S1 to assess the existence of bilayers. The insertion of membrane or soluble proteins in the phase at concentrations below 60 µM does not modify the parameters of the phase (Supplementary data Figure S1). This result is the same for interacting proteins inserted in the sponge phase. Moreover, freeze fracture electron micrographs of the L_3_ phase do not show segregation process in the protein localization and show no change in the L_3_ phase structure after protein incorporation.

### Circular Dichroism (CD) spectroscopy

CD spectra were recorded at 25°C using a JASCO J-810 spectropolarimeter. The far UV CD spectroscopic measurements were carried out in a 1 mm path-length cuvette (110-QS P, from Hellma). For these measurements we used a protein concentration of 50 µM. The secondary structure contents of MexA and OprM were calculated from their far UV CD spectra using the K2D software. For a protein concentration error in sponge phase of less than 4%, we determined that the contents of α-helix and β-sheet of both proteins were similar to that found in their respective crystallographic structures. Furthermore, we observe in Figure S3 (Supplementary data) only small differences between the spectra of the proteins (the transmembrane OprM and the membrane MexA proteins) solubilized in solution (with β-OG) and incorporated into the sponge phase, as observed for other proteins [59]. This shows that the conformation of both proteins is conserved after incorporation into the sponge phase.

### Fluorescence Recovery After fringe Pattern Photobleaching (FRAPP)

Proteins were incorporated into the L_3_ phase at concentrations varying from 0.1 to 5 µM. A fringe pattern was produced by two Argon ion laser beams (Spectra-Physics) set to a wavelength of 488 nm, and focused onto the capillary containing a 10 µL sample. Fringe spacing, *i*, was controlled and varied from 5 to 100 µm. A 50 ms long exposure under a 400 mW laser illumination resulted in the bleaching of the fluorescent molecules located in the bright fringes, whereas the other molecules retained their fluorescence. The diffusion of the fluorescent molecules resulted in the recovery of the initial intensity in the bright fringes that was monitored by a probe laser and collected over a wide area by a photomultiplier. Because the detection was carried out over a large number of bilayers and of proteins, a high signal to noise ratio was achieved. In a single pulse, measurements were actually averaged over 10^8^ proteins. If a single molecule species was diffusing, the recovery signal was characterized by a single exponential decay with a characteristic recovery time *τ* [32]. If the sample contained several molecular species, the recovery intensity was a sum of exponentials (Fig. 1). The recovery time associated with each exponential gave a diffusion coefficient characteristic of one species. The intensity associated with each exponential was also proportional to the associated molecular species. The systematic use of at least 4 interfringe sizes *i* (1 µm<*i*<200 µm) allowed us to check the Brownian diffusion of all peptides, and to obtain the diffusion coefficient from 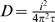, with a precision better than 4%. Five different samples per experiment were used to extract statistics on diffusion coefficients.

## Supporting Information

Figure S1 X-ray spectra of the L3 phase with and without proteins. X-ray spectra realized for a L3 phase of ϕ_m_ = 0.3 with (- -) and without (-) MexA protein. The insert shows a graph of the intensity vs d^2^ for small d values. The results are well-fitted by a d^2^ variation characteristic of bilayer structure.
(0.18 MB TIF)

Figure S2 Freeze fracture electron micrograph of the L3 phase. Freeze-fracture electron microscopy realized for a L3 phase of ϕ_m_ = 0.25.
(0.96 MB TIF)

Figure S3 Ultraviolet CD spectra of proteins MexA and OprM. Far UV CD spectra performed on: (i) lipid anchored membrane protein MexA in a solution of β-OG (- - -(black)) (ii) MexA incorporated into the L3 phase (- - -(grey)), (iii) the transmembrane protein OprM in a solution of β-OG (black solid line) and (iv) OprM incorporated into the L3 phase (grey solid line).
(0.21 MB TIF)
