# Supplementary figures and images for: Tracking Membrane Protein Association in Model Membranes
Source: PLoS One. 2009 Apr 1;4(4):e5035. doi: 10.1371/journal.pone.0005035 (PMC2659767; doi:10.1371/journal.pone.0005035)

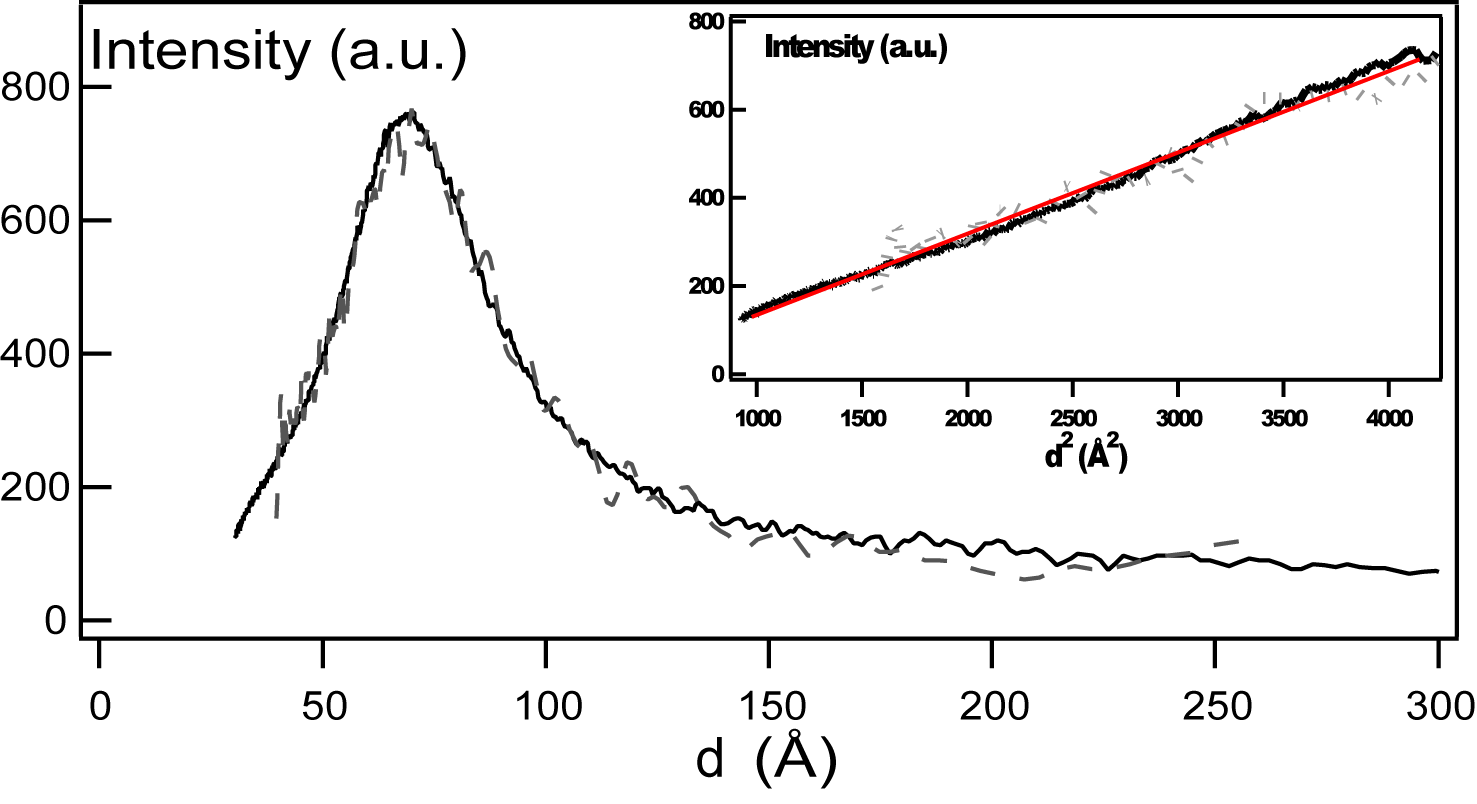

Supplement: Figure S1 — X-ray spectra of the L3 phase with and without proteins. X-ray spectra realized for a L3 phase of ϕm = 0.3 with (- -) and without (-) MexA protein. The insert shows a graph of the intensity vs d2 for small d values. The results are well-fitted by a d2 variation characteristic of bilayer structure. (0.18 MB TIF) [file pone.0005035.s001.tif]

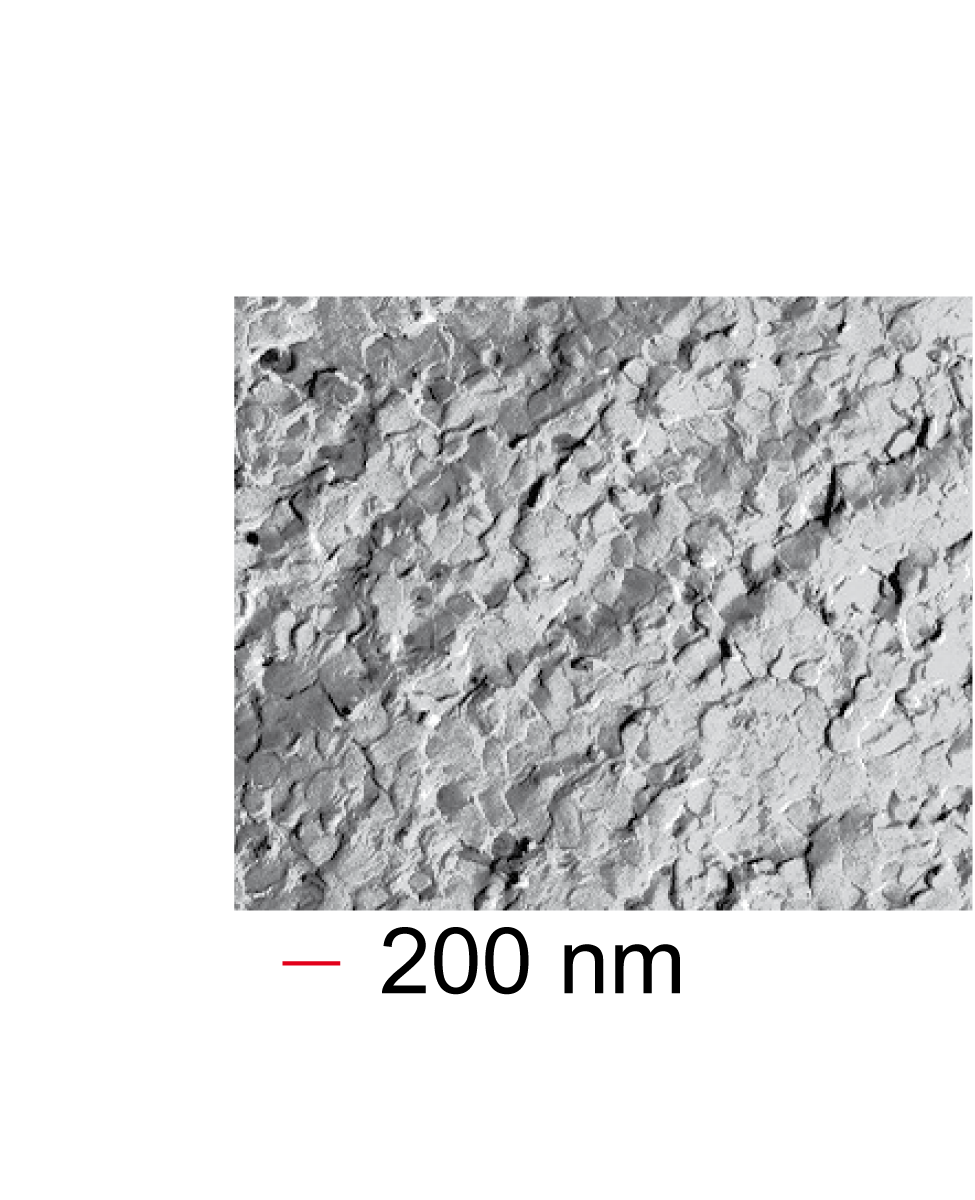

Supplement: Figure S2 — Freeze fracture electron micrograph of the L3 phase. Freeze-fracture electron microscopy realized for a L3 phase of ϕm = 0.25. (0.96 MB TIF) [file pone.0005035.s002.tif]

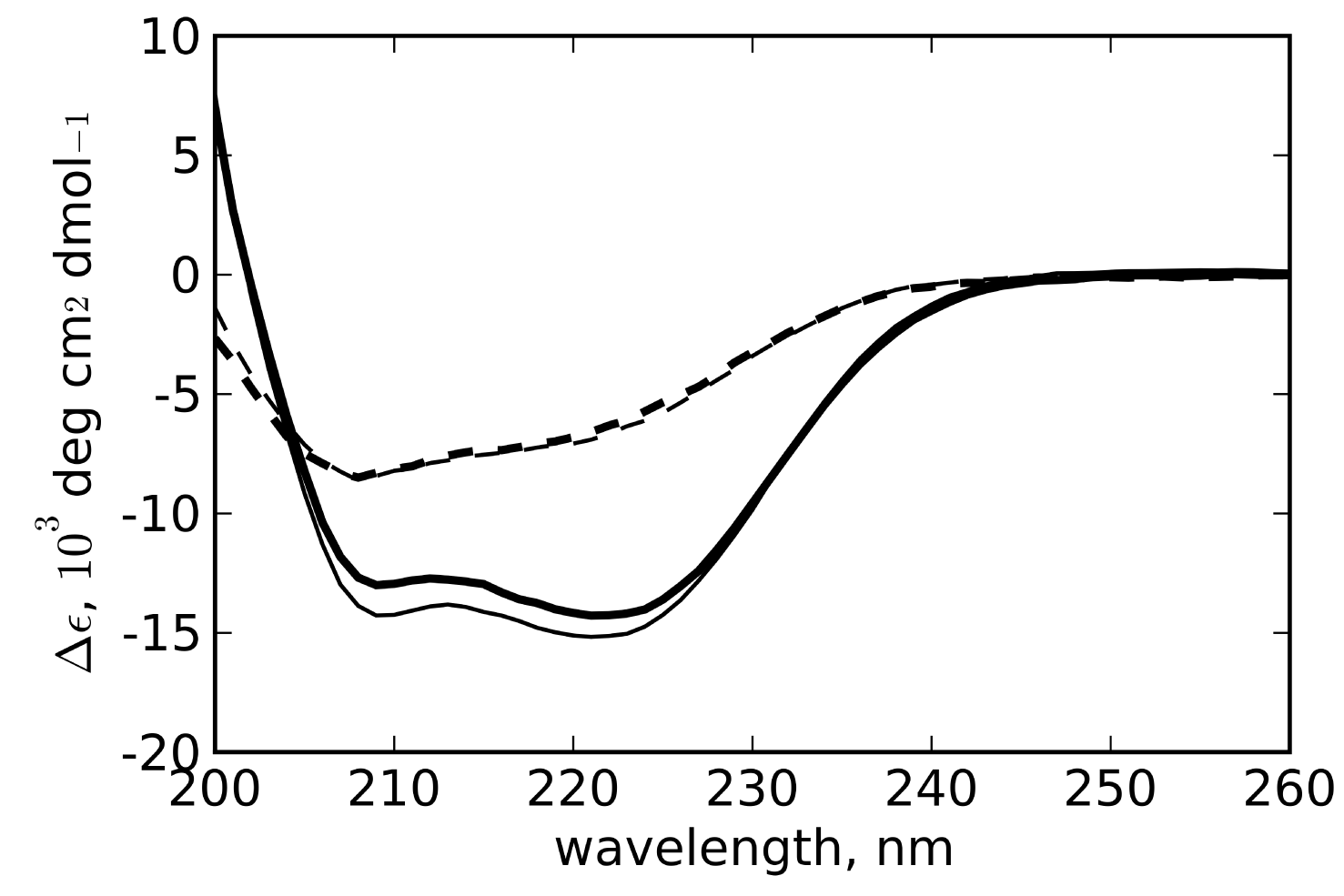

Supplement: Figure S3 — Ultraviolet CD spectra of proteins MexA and OprM. Far UV CD spectra performed on: (i) lipid anchored membrane protein MexA in a solution of β-OG (- - -(black)) (ii) MexA incorporated into the L3 phase (- - -(grey)), (iii) the transmembrane protein OprM in a solution of β-OG (black solid line) and (iv) OprM incorporated into the L3 phase (grey solid line). (0.21 MB TIF) [file pone.0005035.s003.tif]
